# Impact of dexmedetomidine on postoperative cognitive dysfunction and inflammatory response in older adult female patients undergoing laparoscopic cholecystectomy

**DOI:** 10.3389/fsurg.2025.1636040

**Published:** 2025-10-23

**Authors:** Yanlong Fu, Xiaoli Dong, Qiang Wei, Zhenliang Wang, Qingtao Zhao, Wenxin Shi

**Affiliations:** 1 Department of Hepatobiliary Surgery, The 980 Hospital of the Joint Service Support Force of the People’s Liberation Army of China, Shijiazhuang, Hebei, China; 2 North China University of Science and Technology, Caofeidian New Town, Hebei, China; 3 Department of Reproductive Medicine, Longyan First Affiliated Hospital of Fujian Medical University, Longyan, Fujian, China; 4 Department of Obstetrics and Gynecology, Hebei General Hospital, Shijiazhuang, Hebei, China

**Keywords:** dexmedetomidine, postoperative cognitive dysfunction, inflammatory cytokines, postoperative pain, laparoscopic cholecystectomy

## Abstract

**Introduction:**

The aim of the present study was to identify the minimum effective dose of dexmedetomidine (Dex) that could be safely and effectively promoted for clinical application. A rigorous comparison between multiple Dex dosage groups and the control group was conducted.

**Methods:**

Based on the inclusion criteria, 165 elderly patients undergoing LC in our hospital were randomly divided into four groups: Group C (the control group, 32 patients), group D1 (low-dose Dex, 41 patients), group D2 (medium-dose Dex, 49 patients), and group D3 (high-dose Dex, 43 patients). The effects of different doses of Dex on postoperative cognitive impairment, pain scores, and inflammatory markers were subsequently studied in the selected patients.

**Results:**

Patients who received the medium dosage of Dex experienced significantly lower incidences of postoperative agitation and tachycardia compared with the control group, and all doses of Dex reduced the incidence of Postoperative cognitive dysfunction (POCD). It was highlighted the efficacy of medium and high doses of Dex in achieving superior analgesia (as evidenced by lower VAS scores) at different postoperative time points. Concordantly, it was also revealed a similar pattern in postoperative recovery quality. After comparing the low-dose, medium-dose, and high-dose groups with the control group, we found that only the medium-dose group significantly decreased the expression levels of IL-1β and TNF-α both on the first day and second day postoperatively, while the expression levels of IL-10 increased.

**Discussion and Conclusion:**

In conclusion, compared with normal saline, a 0.6 μg/kg/h maintenance dose of Dex is the optimal dosing regimen for improving postoperative cognitive function and had a better anti-inflammation effect in elderly female patients following LC.

## 1 Introduction

Postoperative cognitive dysfunction (POCD) represents a common early syndrome affecting the central nervous system following operation and anesthesia. It was characterized by a decline in cognitive function, including postoperative anxiety, impaired memory, reduced attention, and even personality changes, all of which impede postoperative recovery (1), and is frequently observed in older patients undergoing general surgical procedures. POCD within a very short time after the operation occurred with a different frequency: from 17 to 56% with a tendency to resolve over time (1). Consequently, clinicians and anesthetists are advised to pay significant attention to the onset and preventive measures of POCD.

Dexmedetomidine (Dex), a highly selective *α*2 receptor agonist, possesses anti- inflammatory, sedative, analgesic, anxiolytic, anti- sympathetic, and mild respiratory depression properties. Furthermore, it exerts a preventive effect on POCD after cardiac and non-cardiac surgeries (2) and anesthesia-induced postoperative delirium (3). Yu et al. (4) performed a meta- analysis, which suggested that Dex was associated with a reduced risk of POCD in older adults. Their findings led them to the conclusion that Dex stand out from competitor drugs as having the highest potential to decrease the incidence of POCD in older adults who are undergoing noncardiac surgery (5).

**Figure 1:**
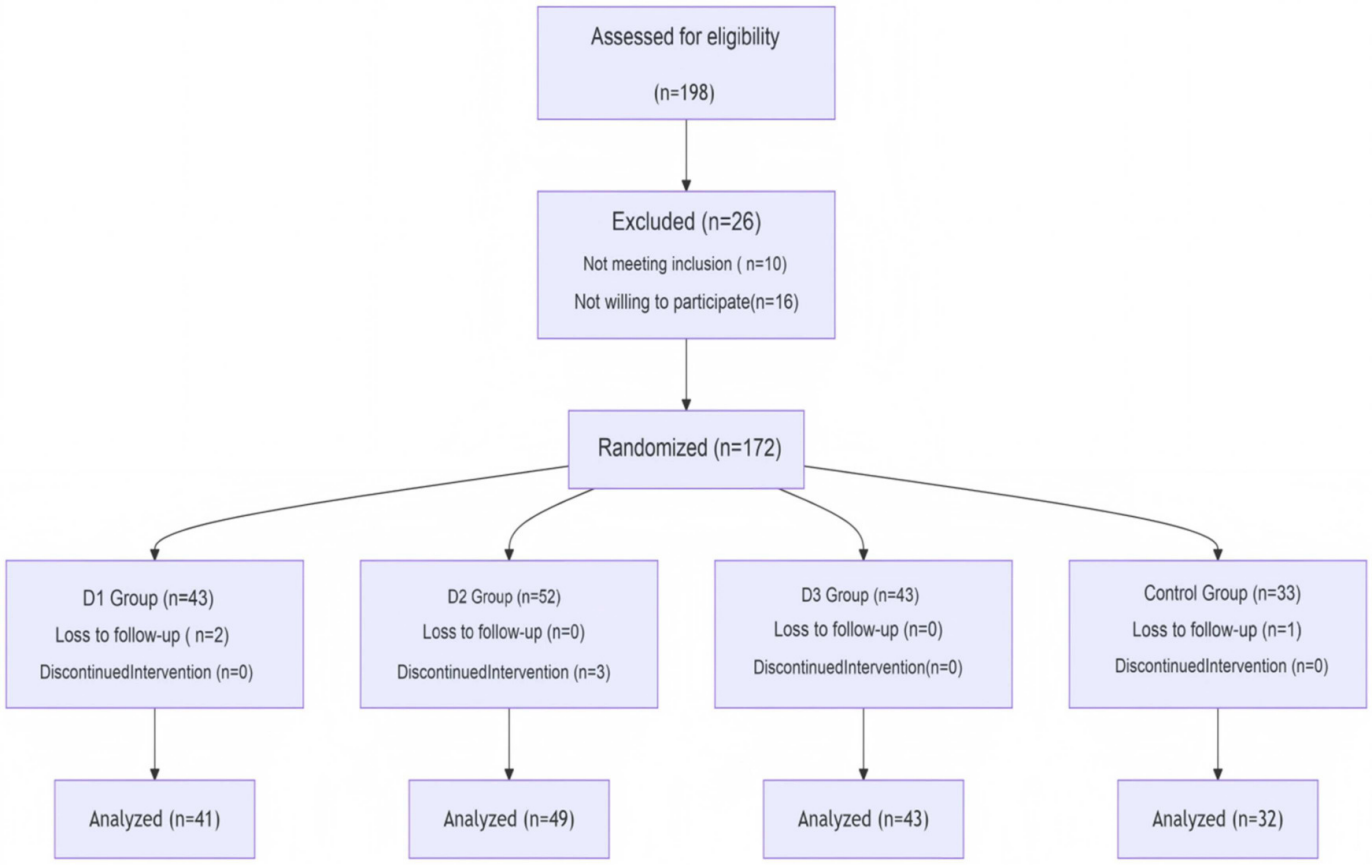
Flow chart of the study selection procedure.

Laparoscopic cholecystectomy (LC) is currently the preferred treatment method for cholecystitis and gallstone disease. Elderly patients are particularly vulnerable to postoperative cognitive dysfunction (POCD), which presents itself as a severe complication of laparoscopic procedures (6, 7). POCD often occurs within the first few weeks following surgery, with an incidence rate of approximately 10%–54% (8). Various factors can trigger postoperative cognitive impairment in patients, including age, low educational level, high-risk surgical types, postoperative complications, postoperative infections, among others. Its occurrence may also be associated with inflammation in the central nervous system inflammation, neurotransmitter abnormalities, among others defects (9). Previous studies had shown that dexmedetomidine (Dex) was able to reduce the incidence of POCD following cardiac (10) and non-cardiac (11–14) surgeries effectively. Previously published literature reviews had primarily focused on POCD in patients undergoing cardiovascular surgery, whereas limited attention has been given to those patients undergoing LC. In particular, to the best of the authors' knowledge, the impact of Dex on cognitive impairment in older adult patients undergoing LC has yet to be explored. Furthermore, no previous studies have examined how different doses of Dex may affect POCD in older adult female patients after having undergone LC. The present study aimed to elucidate the effects of varying doses of Dex on early postoperative cognitive dysfunction (POCD) in older adult female patients undergoing LC. Additionally, it sought to investigate whether the anti-inflammatory properties of Dex mediate this process (Figure 1).

## 2 Patients and methods

### 2.1 General information

This was a prospective randomized double-blind controlled clinical trial. The present study included 165 elderly female patients who had elective general anesthesia for LC at the Joint Logistics Support Force of the People's Liberation Army of China's 980th Hospital between July 2023 and July 2024. Patients in group C received saline, whereas those in group D received a 0.5 µg/kg infusion of Dex infusion 15 min prior to anesthesia induction, followed by postoperative infusions of 0.4, 0.6, and 0.8 µg/(kg·h) Dex to maintain anesthesia. Patients will be assigned to differert groups using the minimization method in the web registration system and issued case numbers. The physicians in charge registered and assigned patients using this system. These patients were further subdivided three groups: D1 (low-dose Dex, *n* = 41), D2 (medium-dose Dex, *n* = 49), and D3 (high-dose Dex, *n* = 43) group C (the control group) received saline, (*n* = 32). Demographic and clinical characteristics of the patients, including age, sex, medical history, laboratory data, pre- and post-operative scores of Mini- Mental State Examination (MMSE) and Montreal Cognitive Assessment Scale (MOCA), was recorded. The inclusion criteria were as follows: (i) the patients were assigned an American Society of Anesthesiologists (ASA) score of I–II; (ii) the female patients were aged 65–75 years; (iii) the pathological diagnosis was of gallstones or cholecystitis, and the patients were undergoing LC; and (iv) a MMSE score >27 was assigned one day prior to surgery. The exclusion criteria were as follows: (i) the pathology reports indicated malignant tumors; (ii) there was a change in surgical procedure during the operation; (iii) the patient was allergic to anesthetic drugs; (iv) the patient had a history of psychiatric illness, or were receiving a corresponding medication for the condition; and (v) the patient had a history of drug abuse or (vi) coexisting internal medical diseases, including hypertension, diabetes, hyperlipidemia, cerebral infarction. The Medical Ethics Committee of our institution approved this study (Ethics Approval Number: 2021-KY-910), either the patients or their families provided informed consent. It was confirmed that all methods were performed in accordance with the relevant guidelines and regulations of the medical ethics committee of our institution. Eligible participants are identified through a joint assessment by anesthesiologists and hepatobiliary surgeons, with written informed consent obtained from patients and their families prior to enrollment.

#### 2.1.1 Sets of patients

Routine preoperative preparations were made, and general anesthesia was employed. Venous access was established, accompanied by concurrent cardiac monitoring, blood oxygen saturation, and blood pressure monitoring, as well as bispectral index monitoring of brain electrical activity. Sufentanil (0.3 μg/kg), rocuronium bromide (0.6 mg/kg), and 1% propofol injection (2.0–2.5 mg/kg) were used for the induction of anesthesia. Following endotracheal intubation, injection of 1% propofol injection at 4–5 mg/(kg·h), infusion of remifentanil at 0.1–0.2 μg/(kg·h) of remifentanil infusion, and 2%–3% sevoflurane inhalation were used to maintain anesthesia. Patients in groups D1- D3 received an intravenous infusion of 0.5 μg/kg Dex within 15 min prior to the induction of general anesthesia, followed by maintenance doses of 0.4, 0.6, and 0.8 μg/(kg·h) Dex, respectively; by contrast, group C received an equivalent volume of saline. Throughout this period, the bispectral index remained between 45 and 60. Mechanical ventilation was used, while maintaining the keeping peak airway pressure at a level <25 cm H_2_O, and the end- tidal CO_2_ pressure was maintained between 37 and 45 mmHg.

#### 2.1.2 Assessment indicators

Patient demographics, including age, education level, body mass index (BMI), ASA classification, and surgery duration were also collected. Heart rate changes and intraoperative hypotension were compared across the four groups (i.e., groups C and D1-D3).

#### 2.1.3 Cognitive function assessment

The MMSE was performed prior to inducing anesthesia, induction and on the first, second, and third postoperative days to assess the presence of POCD in patients. The following were the MMSE scoring criteria: Normal cognition was defined as 28–30 points, mild POCD as 24–27 points, moderate POCD as 19–23 points, and severe POCD as 0–18 points (8). Each group's number of POCD cases was recorded, and a reactions in every group (nausea, agitation, tachycardia, bradycardia, and postoperative somnolence) were observed.

The visual analog scale (VAS) scores in a resting state on days 1, 2, and 3 post-surgery were recorded. The VAS scores were in a scale of 0 to 10, with the higher scores signifying more severe pain. The Athens insomnia scale (AIS) scores were recorded one day prior to surgery, and on days 1, 2, and 3 postoperatively. On days 1, 2, and 3 following surgery, central venous blood (4 ml) was collected and serum was centrifuged (3,500–4,000 rpm for 5 min,at room temperature) to quantify the levels of interleukin IL- 1β, IL- 10, and tissue necrosis factor TNF- ɑ levels using the enzyme-linked immunosorbent assay.

#### 2.1.4 Criteria for discontinuing or modifying allocated interventions

The investigator will terminate the experiment for this participant if one of the following occurs during the experiment: (1) Those who have difficulty maintaining intraoperative blood pressure and heart rate and those who experience serious intraoperative complications (hemorrhagic and anaphylactic shock, cardiac arrest, etc.). (2) Patients who refuse neurological test scores after surgery. (3) The investigator may decide to end the trial due to other unforeseen reasons.

#### 2.1.5 Statistical methods

The sample size calculation was based on the incidence of POCD in elderly patients during operation and calculated using the PASS 20.0 software (IBM Corp.). According to a previous study (13), we selected the incidence of POCD that required a larger sample size, and the sample size was calculated to be 30 (power = 0.8, α = 0.05). In our study, we enrolled more than 30 patients per group that greater than the expected number of subjects.

Group comparisons were performed using the Fisher's exact test, and quantitative results were shown as the mean ± standard deviation. Intergroup comparisons were performed using the one-way Analysis of Variance (ANOVA) and Kruskal–Wallis test, and the Dunnett's/Dunn's *post hoc* tests are used for the pairwise comparisons, with the counted data presented as the number of cases and as percentages (%). *P* < 0.05 was considered to indicate a statistically significant difference.

## 3 Results

### 3.1 General data

The age, education level, BMI values, ASA scores classification, and surgery duration data of the patients were found not to differ significantly among the four groups (*P* > 0.05; see Table 1).

**Table 1:** Characteristics of patients underwent laparoscopic cholecystectomy among the four groups.

Groups	Age (year)	Education (year)	BMI (kg•m^−2^)	Length of surgery (mim)	ASA status *n*(%)	
					Ⅰ	Ⅱ
Group C	67.72 ± 6.41	7.8 ± 1.2	21.98 ± 6.78	95.05 ± 23.7	19 (59.4)	13 (40.6)
Group D1	68.23 ± 7.14	7.4 ± 2.6	24.08 ± 1.17	92.08 ± 35.6	22 (53.7)	19 (46.3)
Group D2	69.25 ± 5.32	7.3 ± 4.7	23.18 ± 4.08	91.05 ± 41.7	22 (44.9)	27 (55.1)
Group D3	68.56 ± 8.19	7.7 ± 1.3	22.92 ± 2.55	94.61 ± 19.08	24 (55.8)	19 (44.2)
*P*	0.231	0.354	0.159	0.258	0.691	0.965

Values were presented as mean ± standard deviation or number (percentage).

Incidence of Postoperative Cognitive Dysfunction (POCD) and Adverse Reactions.

Upon comparing the incidence of POCD was compared between the Dex and saline treatment groups, it was found that the low, medium, and high doses of Dex [0.4, 0.6 and 0.8 μg/(kg·h) Dex, respectively] all effectively the incidence of POCD (*P* < 0.05). In terms of additional postoperative adverse responses, the incidence of postoperative agitation and tachycardia were found to be statistically significantly reduced in D2 group compared with group C (*P* < 0.05); however, no significant differences were identified in terms of the other adverse reactions (Table 2).

**Table 2:** Comparison of the incidence of postoperative cognitive dysfunction and other postoperative side effects. [*n* (%)].

Groups	POCD	Postoperative nausea	Postoperative agitation	Postoperatively tachycardia	Postoperative bradycardia	Postoperative drowsiness
Group C	6 (18.75%)	4 (12.5%)	5 (15.625%)	8 (25%)	4 (12.5%)	2 (6.25%)
Group D1	2 (4.89%)*	3 (7.32%)	5 (12.20%)	5 (12.20%)	3 (7.32%)	2 (4.89%)
Group D2	3 (6.12%)*	3 (6.12%)	3 (6.12%)*	4 (8.16%)*	2 (4.08%)	1 (2.04%)
Group D3	1 (2.33%)*	2 (4.65%)	4 (9.30%)	6 (13.95%)	4 (9.30%)	2 (4.65%)
*P*	0.016	0.269	0.019	0.029	0.502	0.257

Fisher's exact test were used to compare postoperative side effects between four groups. Data were presented as *n* (%). Compared with Group C.

* *P* < 0.05.

### 3.2 Comparison of the patients' VAS scores

A comparison of the postoperative VAS scores at various time intervals across the four groups revealed that patients in groups D2, and D3 had significantly lower VAS scores on the first day, on the second and third postoperative days compared with those in group C. However, there was no significant differences were identified in terms of the VAS scores for Dex in group D1 on the second and third postoperative days compared with the saline group (Table 3).

**Table 3:** Comparison of visual analog scale scores at various time according to the study groups.

Groups		1 day postoperatively	2 days postoperatively	3 days postoperatively
Group C	Mean ± SD	2.43 ± 0.31	3.29 ± 0.81	1.99 ± 0.73
	Median [IQR]	2.34 [1–3]	3.45 [1–5]	2.01 [1–3]
Group D1	Mean ± SD	1.84 ± 1.09*	2.57 ± 1.72	1.83 ± 0.81
	Median [IQR]	2 [1–3]	2 [1–4]	1 [1–3]
Group D2	Mean ± SD	1.80 ± 1.09*	2.17 ± 1.56*	1.69 ± 0.42*
	Median [IQR]	1 [1–4]	2 [1–4]	3 [1–3]
Group D3	Mean ± SD	1.72 ± 1.01*	2.04 ± 1.35*	1.25 ± 2.23*
	Median [IQR]	2 [1–3]	1 [1–3]	1 [1–3]
*P*		0.025	0.018	0.044

Data were presented as mean ± standard deviation. Compared with Group C.

* *P* < 0.05.

### 3.3 Comparison of the patients' AIS scores

A comparative analysis of the AIS scores revealed that administering medium and high doses of Dex resulted in a significant reduction in the incidence of postoperative insomnia across all the measured time intervals compared with the control group (group C). In addition, the group administered a low dose of Dex (i.e., group D1) showed no significant reduction in postoperative insomnia with the control group (*P* < 0.05; Table 4).

**Table 4:** The differences of Athens insomnia scale scores according to the study groups.

Groups		1 day before surgery	1 day postoperatively	2 days postoperatively	3 days postoperatively
Group C	Mean ± SD	3.71 ± 1.20	8.21 ± 6.72	6.69 ± 3.61	4.81 ± 1.63
	Median [IQR]	5 [2–6]	8 [4–10]	6 [4–9]	5 [4–8]
Group D1	Mean ± SD	3.82 ± 2.30	5.24 ± 3.28	4.54 ± 1.92	3.49 ± 2.01
	Median [IQR]	4 [2–7]	5 [4–10]	4 [3–6]	3 [2–6]
Group D2	Mean ± SD	3.59 ± 2.92	5.01 ± 0.86*	4.33 ± 1.09*	3.23 ± 2.87*
	Median [IQR]	5 [2–8]	6 [4–10]	5 [3–6]	3 [1–5]
Group D3	Mean ± SD	3.25 ± 1.79	4.01 ± 0.72*	3.01 ± 4.22*	3.04 ± 0.65*
	Median [IQR]	4 [2–7]	4 [2–8]	3 [1–5]	2 [1–4]
*P*		0.651	0.048	0.036	0.025

Data were presented as mean ± standard deviation. Compared with Group C.

* *P* < 0.05.

### 3.4 Comparison of inflammatory markers in patients

The evaluation of serum inflammatory markers on the initial and subsequent days following surgery revealed that, for Group D2 and Group D3, the concentrations of IL-1βand TNF-αwere significantly reduced compared with the control group (Group C). Furthermore, the concentrations of IL-10 were significantly increased (*P* < 0.05; Table 5).

**Table 5:** The differences of inflammatory markers according to the study groups.

Groups	IL-1β (pg/ml)		IL-10 (pg/ml)		TNF-α (pg/ml)	
	1 day postoperatively	2 days postoperatively	1 day postoperatively	2 days postoperatively	1 day postoperatively	2 days postoperatively
Group C	279.89 ± 27.54	177.81 ± 18.59	35.06 ± 0.93	59.04 ± 8.64	249.77 ± 9.83	190.51 ± 9.59
Group D1	192.01 ± 23.98	113.23 ± 32.18	52.93 ± 2.79	72.13 ± 9.92	208. ± 31.01	167.98 ± 10.69
Group D2	180.98 ± 34.01*	110.71 ± 19.80*	50.28 ± 2.05*	99.18 ± 9.89*	185.62 ± 21.90*	154.56 ± 20.18*
Group D3	171.63 ± 45.82*	101.49 ± 37.74*	57.62 ± 31.01*	79.57 ± 12.23	176.83 ± 31.74*	132.55 ± 2.84*
*P*	0.026	0.014	0.043	0.039	0.007	0.018

Data were presented as mean ± standard deviation. Compared with Group C.

* *P* < 0.05.

## 4 Discussion

This study demonstrates that intraoperative infusion of dexmedetomidine (Dex), particularly at medium [0.6 μg/(kg·h)] and high [0.8 μg/(kg·h)] doses, confers significant benefits in the postoperative period compared to a saline control. The findings indicate that these doses are effective in reducing the incidence of POCD, alleviating pain and insomnia, mitigating certain adverse reactions, and modulating the surgical stress response by attenuating pro-inflammatory cytokines while enhancing anti-inflammatory activity.

The comparable baseline characteristics and operative duration across all groups suggest that the observed differences are likely attributable to the intervention rather than patient or procedural disparities. Notably, a significant reduction in POCD incidence was observed across all Dex dosage levels, which is consistent with the established neuroprotective properties of Dex. It potentially mediated via α2-adrenoreceptor agonism, leading to anti-apoptotic effects, attenuation of neuroinflammation, and preservation of cerebral perfusion (15, 16).

The number of geriatric patients with gallbladder diseases is progressively increasing in tandem with the prevalence of aging; therefore, complications following LC warrant greater attention (8). POCD is one of the complications of LC, having a relatively high incidence rate (8). Both understanding the underlying mechanisms of the development of POCD and preventing its occurrence should be assigned adequate importance. Recent studies have shown that surgery- induced neuroinflammation is a significant contributor towards POCD induction (15, 16). Surgery or trauma typically leads to an increased production of systemic pro-inflammatory factors, triggering neuroinflammation, which subsequently causes neuronal dysfunction and a decline in cognitive abilities (17, 18). On the other hand, inhibiting neuroinflammation may also lead to an improvement in cognitive dysfunction and hinder the occurrence and progression of POCD (19).

As an α2- adrenergic receptor agonist, Dex has sedative, analgesic, anxiolytic, and sympathetic blocking effects. Due to its ability to enhance neural blockade and shorten the onset time of local anesthetics, it has been widely used in clinical practice (20–23). Dex also possesses certain anti- inflammatory properties (24) and has been shown to reduce stress responses and protect cognitive function through inhibiting inflammatory reactions, effectively preventing the occurrence of POCD (25). This effect may be associated with two mechanisms: (i) Dex may inhibits the sympathetic nervous system by acting on α2-adrenergic receptors, thereby reducing stress and inflammatory responses (26); and (ii) Dex may reduce the expression of inflammatory factors by inhibiting certain signaling pathways (27, 28). Pro-inflammatory factors including TNF- α, IL- 1β, and the anti-inflammatory factor IL- 10 are major inflammatory markers during the perioperative period, and their expression levels can be used to reflect the severity of the inflammatory response. The results obtained in the present study have shown that, in the three Dex treatment groups, reductions were noted in the expression of pro-inflammatory factors (TNF- α and IL- 1β) in the postoperative serum, although the expression level of the anti-inflammatory factor (IL- 10) was increased. These findings were in agreement with those reported in the study of Duan et al. (29). Therefore, we consider that treating patients with different doses of Dex may have certain anti-inflammatory effects, however, the dosages of administered Dex vary, and there are no recommended clinical dosages. In the present study, low, medium, and high dosages of Dex [i.e., 0.4, 0.6 and 0.8 μg/(kg·h) Dex, respectively] all led to decreases in incidence of POCD (*P* < 0.05), which is consistent with the findings of VAN (30). Therefore, our hypothesis is that Dex is able to both reduce the incidence of POCD in elderly patients undergoing LC and enhance postoperative cognition. However, Turan et al. (31) argued that the perioperative use of Dex does not lead to significantly improvements in postoperative cognition in the elderly, and that this may be associated with the age of the study subjects, the dosage and timing the administration of Dex administration, and the lack of a loading dose. By contrast, our trial used a loading dose method, achieving a steady-state plasma concentration at an earlier stage, which enabled the advantages of Dex to be exploited more fully.

Dex is a commonly used sedative in clinical settings, effective in alleviating perioperative stress responses and increasing patient comfort during the perioperative period (23). Dex works by acting on the sympathetic nervous system, inhibiting its excitability, blocking pain signal transmission, and reducing bodily stress caused by factors such as pain and hemodynamic fluctuations (32). Dex may be administered intravenously and used locally, such as in the case of thoracic paravertebral block (33) and is known for its excellent analgesic effects (34). Within three days of surgery, the VAS scores of patients in groups D2 and D3 were found to be substantially lower than compared with those in group C. Therefore, it may be inferred that optimal doses of Dex effectively relieves postoperative pain and improves patient comfort. The results of the present study have also shown that the incidence of postoperative agitation and tachycardia in group D2 was significantly lower compared with group C. By comparing the AIS scores, we have determined that low-dose Dex is associated with a decreased incidence of postoperative insomnia during the initial postoperative phase.On the other hand, medium and high doses of Dex are able to maintain a sustained reduction in the incidence of postoperative insomnia. Taken together, the present study has shown that medium doses of Dex can effectively reduce POCD and other postoperative adverse reactions. In conclusion, a medium maintenance dose of 0.6 μg/(kg·h) of Dex may significantly reduce postoperative pain and the occurrence of adverse reactions such as POCD following LC surgery, demonstrating high safety and being worthy of clinical application.

One limitation of the present study is that, although the effects of three different dosages of Dex on early postoperative cognitive impairment in elderly patients receiving LC were investigated, no long- term follow- up or surveillance was performed. Furthermore, the study did not delve deeply into the mechanisms underlying POCD development. It only revealed that this process may be associated with inflammation mediated by certain inflammatory factors, but there was no extensive exploration of whether it is associated with specific signaling pathways, and this necessitates further research on our part.

In conclusion, medium dosage of Dex appeard to offer a favorable balance between minimizing adverse reactions, enhancing analgesia, improving recovery quality, and modulating the inflammatory cascade. In elderly female patients following LC, a medium maintenance dose of 0.6 g/(kg·h) of Dex has been shown to minimize the occurrence of early POCD. This effect is likely to be associated with Dex's ability to mediate inflammatory responses. Additionally, this dosage of Dex may reduce certain postoperative complications, providing a reference for clinical medication.

## Data Availability

The original contributions presented in the study are included in the article/Supplementary Material, further inquiries can be directed to the corresponding author.
